# NUA and ESD4 negatively regulate ABA signaling during seed germination

**DOI:** 10.1007/s44154-022-00062-1

**Published:** 2022-09-13

**Authors:** Xiaona Cui, Mengyang Lv, Yuanyuan Cao, Ziwen Li, Yan Liu, Zhenzhen Ren, Hairong Zhang

**Affiliations:** 1 College of Life sciences, Henan Agricultural University, Zhengzhou, 450002 China; 2 State Key Laboratory of Wheat and Maize Crop Science, Henan Agricultural University, Zhengzhou, 450002 China

**Keywords:** ABA signaling, SnRK2, SUMOylation, NUA, ESD4

## Abstract

The phytohormone abscisic acid (ABA) plays important roles in plant growth, development and adaptative responses to abiotic stresses. SNF1-related protein kinase 2s (SnRK2) are key components that activate the ABA core signaling pathway. NUCLEAR PORE ANCHOR (NUA) is a component of the nuclear pore complex (NPC) that involves in deSUMOylation through physically interacting with the EARLY IN SHORT DAYS 4 (ESD4) SUMO protease. However, it is not clear how NUA functions with SnRK2 and ESD4 to regulate ABA signaling. In our study, we found that *nua* loss-of-function mutants exhibited pleiotropic ABA-hypersensitive phenotype. We also found that ABA-responsive genes remarkably up-regulated in *nua* by exogenous ABA. The *nua snrk2.2 snrk2.3* triple mutant and *nua abi5* double mutant partially rescued the ABA-hypersensitive phenotype of *nua*, thereby suggesting that NUA is epistatic to SnRK2s. Additionally, we observed that *esd4-3* mutant was also ABA-hypersensitive. NUA and ESD4 were further demonstrated to physically interact with SnRK2s and negatively regulate ABA signaling by reducing SnRK2s stability. Taken together, our findings uncover a new regulatory mechanism that can modulate ABA signaling.

**Supplementary Information:**

The online version contains supplementary material available at 10.1007/s44154-022-00062-1.

## Introduction

The phytohormone ABA plays critical roles in regulating plant growth, development, and stress responses to abiotic stresses such as drought, salt, and cold (Cutler et al. 2010). The core ABA signaling pathway consists of three major components: PYRABACTIN RESISTANCE1 (PYR1)/PYR1-LIKE (PYL)/REGULATORY COMPONENTS OF ABA RECEPTORS (RCAR) ABA receptors, protein phosphatases 2C (PP2Cs), and SnRK2 protein kinases. In the presence of ABA, PYR1/PYL/PCAR bind with ABA and inhibit the activity of PP2Cs, thus relieving PP2C-mediated inhibition of SnRK2s. The SnRK2 kinases phosphorylate and regulate the activity of downstream components, such as ABI5 and MYB30 transcription factors, which lead to changes in gene expression (Fujii and Zhu 2009; Ma et al. 2009).

The SnRK2 protein kinase family consists of 10 members (SnRK2.1 to 2.10) in Arabidopsis. SnRK2.2, SnRK2.3 and SnRK2.6/Open Stomata 1 (OST1) are the three key members in this kinase family, and act redundantly in the ABA regulation of guard cells, seed germination and seedling growth. The *snrk2.6* mutant cannot induce ABA-stomatal closure (Mustilli et al. 2002), and the *snrk2.2 snrk2.3* double mutant is insensitive to ABA during seed germination and seedling growth (Fujii et al. 2007). The *snrk2.2/2.3/2.6* triple mutant exhibits extreme drought stress sensitivity and highly insensitive to ABA during seed development (Fujii and Zhu 2009; Fujita et al. 2009; Nakashima et al. 2009).

Since SnRK2s function as pivotal positive regulators of ABA signaling, they are tightly regulated to help plants respond to ABA and abiotic stresses. Up till now, a number of studies has focused on the role of SnRK2s phosphorylation modification. Disinhibition of PP2Cs is insufficient to activate SnRK2s (Cai et al. 2014; Wang et al. 2018a, b, c), as some kinases such as brassinosteroid insensitive 2 (BIN2), abiotic stress-responsive Raf-like kinase (ARK) and Raf-like kinases (RAFs) are also required for SnRK2s activation (Saruhashi et al. 2015; Wang et al. 2018a, b, c; Lin et al. 2020; Takahashi et al. 2020). Although the role of SnRK2s’ phosphorylation modification has been extensively studied, mechanisms that can regulate their stability remain elusive. Recently, the stability of SnRK2 proteins regulated via ubiquitylation has been studied. Arabidopsis phloem protein 2-B11 (AtPP2-B11), which is part of a SKP1/Cullin/F-box E3 ubiquitin ligase complex, negatively regulates plant responses to ABA through promoting the degradation of SnRK2.3 (Cheng et al. 2017). SnRK2.6 is ubiquitinated by HIGH OSMOTIC STRESS 15 (HOS15), another E3 ubiquitin ligase (Ali et al. 2019). Proteomic analysis also found that SnRK1.1 and SnRK2.4 were the targets of ubiquitination (Kim et al. 2013). Although ubiquitin proteasome-mediated degradation of SnRK2s has been identified for regulating ABA responses, the underlying mechanism of how SnRK2s degradation is being triggered remains unknown. The post-translation modifications of SnRK2s for balancing ubiquitylation still needs to be investigated.

The nuclear pore complex (NPC) is a large multiprotein complex that is essential for macromolecular trafficking between the cytoplasm and nucleus in the eukaryotic cells. NUCLEAR PORE ANCHOR (NUA), the Arabidopsis homolog of vertebrate Tpr (Translocated Promoter Region), is a nuclear pore protein that is attached to the inner side of the NPCs. NUA physically interacts with EARLY IN SHORT DAYS 4 (ESD4), a small ubiquitin-related modifier (SUMO) protease, to regulate SUMO-homeostasis in Arabidopsis (Jacob et al. 2007; Xu et al. 2007). SUMO is an ubiquitin-like peptide that is conjugated to target proteins. SUMOylation is a type of post-translational modification, that regulates diverse biological processes including flowering, environmental stress, plant pathogen interactions, cell growth and development (Park et al. 2011). SUMOylation occurs in a series of biochemical steps: E1-activation, E2-conjugation, and E3-ligation. SUMO proteins are first processed by SUMO proteases that truncate a fragment of about ten amino acids long from the C terminus, leaving two glycine residues at the end of the mature SUMO protein. The SUMO activating enzyme E1 (SUMO-activating enzyme/SAE) catalyzes ATP-dependent activation of mature SUMO, and transfers it to the SUMO E2 conjugating enzyme (SCE1). Then, SUMO E3 ligases transfer SUMO to the protein substrates (Geiss-Friedlander and Melchior 2007).

SUMOylation is a reversible process, which is mediated by SUMO proteases, through their isopeptidase activity (Hickey et al. 2012; Yates et al. 2016). No study to date has investigated the function of NUA and its partner ESD4 SUMO proteases in the ABA signaling pathway. In our study, we found that the *nua* and *esd4* mutants are hypersensitive to ABA during seed germination and seedling growth. Introduction of the *snrk2.2 snrk2.3* or *abi5* mutation can suppress the ABA hypersensitivity of *nua*, indicating that NUA acts upstream of SnRK2s in ABA signaling. Furthermore, we detected that NUA physically interacts with SnRK2.2, SnRK2.3, and SnRK2.6; regulating their stability. However, physical interactions between ESD4 and SnRK2s were found to be weaker than those in NUA- SnRK2s, suggesting that NUA can act as an adaptor that links ESD4 proteases and SnRK2s. Our findings thereby revealed a new regulatory mechanism that involves NUA, ESD4 and SnRK2 kinases to mediate ABA signaling.

## Results

### NUA negatively regulates the ABA signaling pathway

To investigate the role of NUA in ABA signaling, two T-DNA insertion mutants *nua-2* (SALK_069922) and *nua-3* (SAIL_505_H11) were obtained. The developmental defects of *nua-3* were observed to be more severe than those of *nua-2* since *nua-3* was a stronger loss-of-function NUA allele with T-DNA insertion in the exon (Xu et al. 2007).

**Fig. 1:**
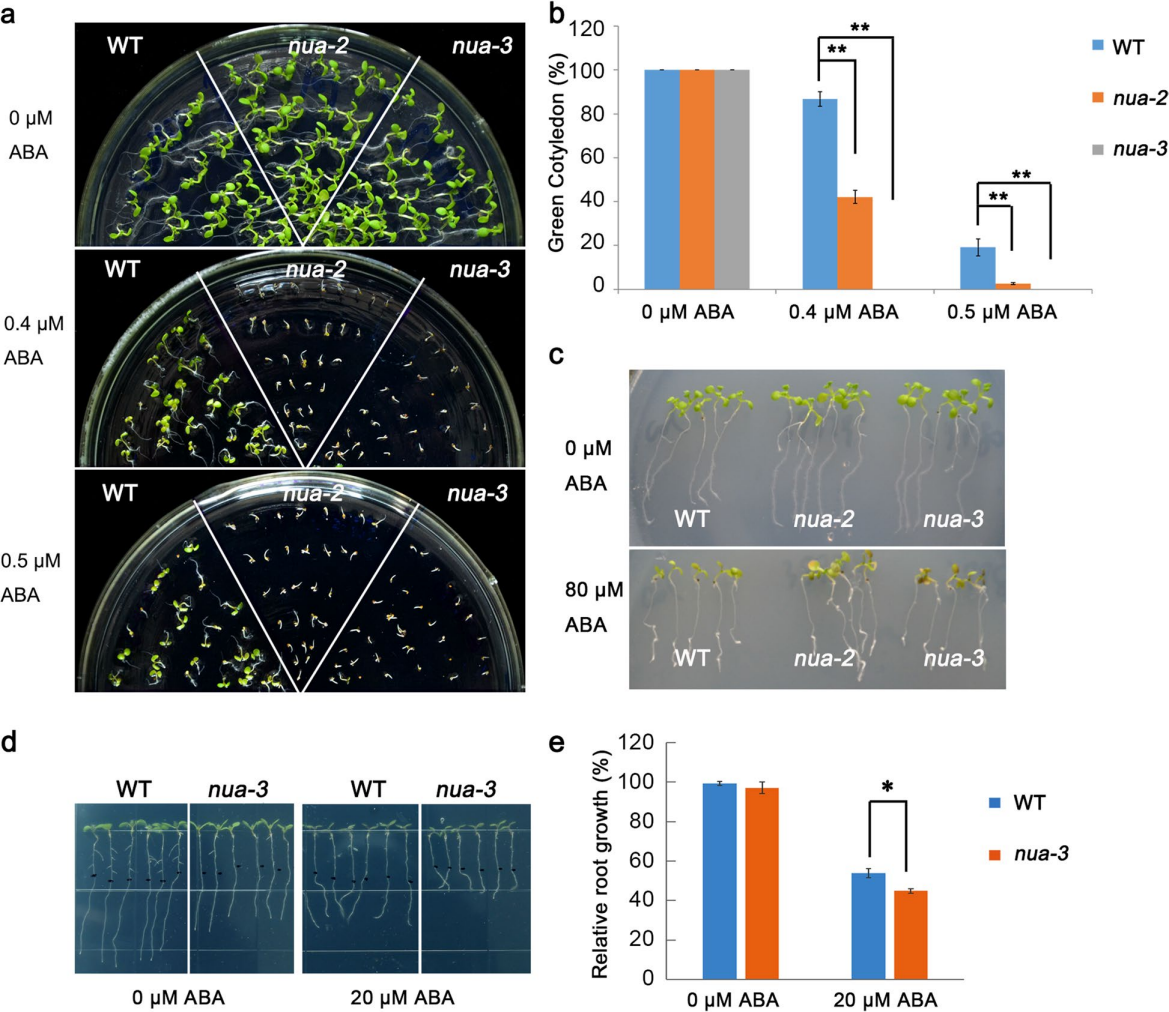
The *nua* mutants are more sensitive to ABA than the wild type. **A** Comparisons of cotyledon greening on MS medium, or MS medium supplemented with different concentrations of ABA between wild type (WT) and two *NUA* loss-of-function mutant alleles. **B** Statistical analyses of green cotyledon ratios. The ratios were counted at Day 5 after the end of vernalization. At least 30 seeds were counted in each replicate. Data are shown as means ± SD of three independent experiments. ** *P* < 0.01 (Student's t-test). **C** The post-germination phenotypes of WT and *nua* mutants on MS containing ABA. Seeds were germinated on MS medium and grown for 4 d before being transferred to MS medium containing 80 μM ABA. The pictures were taken 7 days after transfer. **D** Root growth phenotypes of the WT, *nua-2* and *nua-3* on MS, or MS containing ABA. Seeds were germinated on MS medium and grown for 5 d before being transferred to MS medium or MS medium containing 20 μM ABA. The pictures were taken 5 days after transfer. **E** Statistical analysis of relative root growth of *nua-3* and the WT in (**D**), the elongation root length of the WT and *nua-3* on MS medium was set to 100%. Data are shown as means ± SD of three independent experiments. * *P* < 0.05 (Student's t-test)

We next investigated the effects of ABA treatment on *nua* mutants at the seed germination stage. Without ABA treatment, the green cotyledon ratios of *nua-2* and *nua-3* mutants were observed to be similar as the wild type (WT) at Day 5 after the end of vernalization. In the presence of ABA, however, the *nua* mutants were noted to be more sensitive to the treatment (Fig. 1a). With increasing concentrations of ABA in the MS medium, the cotyledon greening and green cotyledon ratios of *nua* at the seed germination stage exponentially reduced relative to the WT. (Fig. 1a, b).

We further analyzed the phenotype of *nua* mutants in response to ABA during the post germination stage. The 5-day-old seedlings on MS medium were transferred to MS medium containing different concentrations of ABA. Five days later, the primary root length elongation was measured. Our results showed that ABA inhibits primary root growth to a greater extent in *nua-3* than in WT plants (Fig. 1d, e). In addition to affecting root growth, we also noticed that the shoot development of *nua* was hindered in the presence of ABA (significant growth inhibition and chlorotic) compared to the WT (Fig. 1c). Moreover, *nua-3* was less sensitive to salt stress than the wild type, suggesting that *NUA* might also be involved in ABA-dependent salt-stress signal pathways (Fig. S1).

We also tested the relative expression levels of the ABA-responsive genes in *nua* mutant upon ABA treatment. The expressions of *ABI5*, *RD29A* and *RD29B* were remarkably induced by exogenous ABA treatment more than that in the WT (Fig. S2). Taken together, our results indicate that *NUA* is a negative regulator of ABA signaling.

### NUA is epistatic to SnRK2s and ABI5 in ABA signaling

**Fig. 2:**
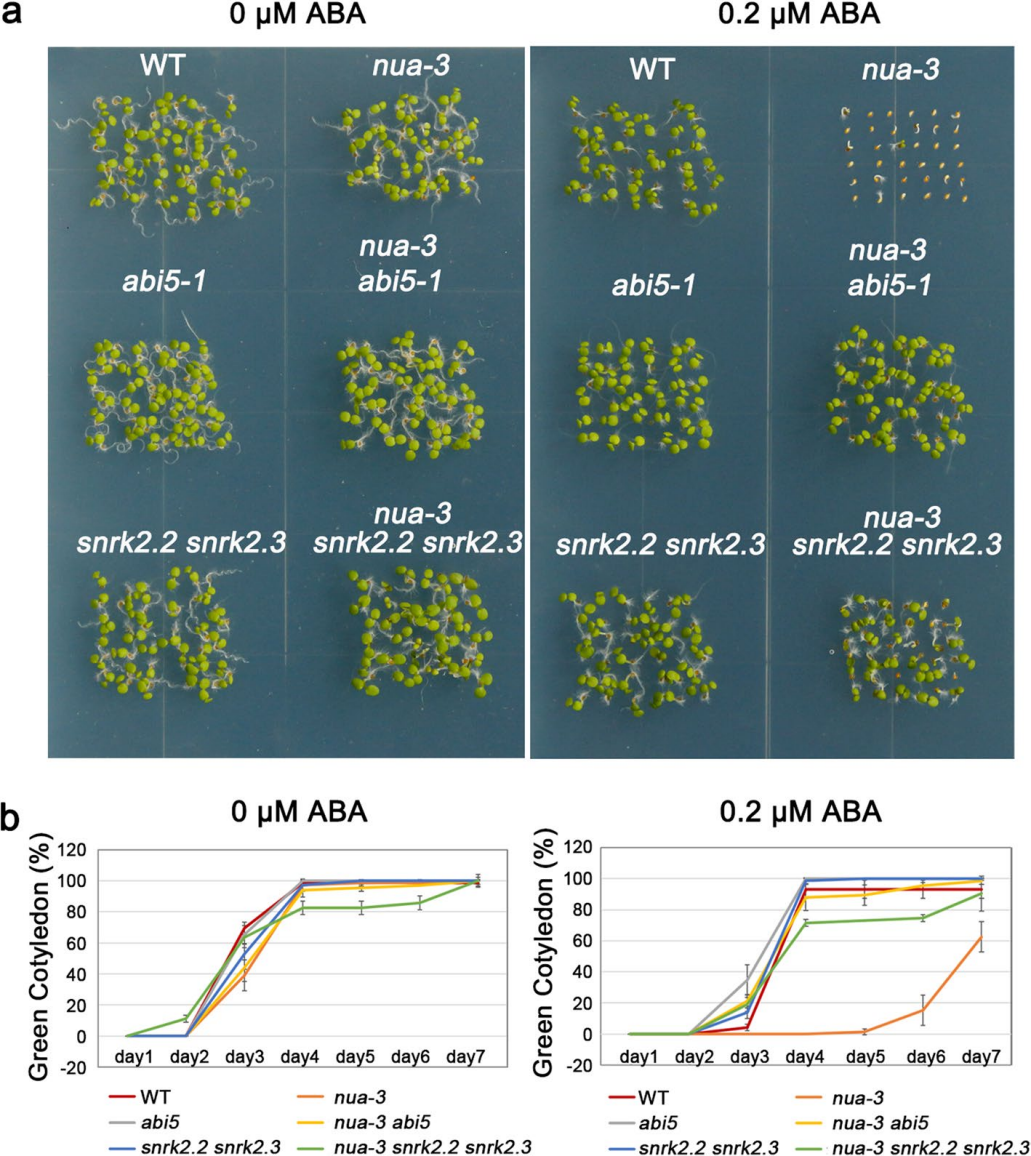
NUA is epistatic to SnRK2.2, SnRK2.3 and ABI5 in controlling seed germination in response to ABA. **a** Seed germination phenotypes. The photos were taken at 5 d after the end of vernalization. **b** Quantitative analysis of the greening rates. Data are shown as means ± SD of three independent experiments

To further elucidate how NUA functions in the ABA signaling pathway, we crossed *nua* with genes involved in ABA signaling and analyzed their interactions. It has been known that *snrk2.2 snrk2.3* and *abi5* are insensitive to ABA during the seed germination stage (Fujii and Zhu 2009). We thus isolated *nua-3 snrk2.2 snrk2.3* triple mutants and *nua-3 abi5* double mutants for studying their responses to ABA during the cotyledon greening stage. In the absence of ABA, the cotyledon greening rates of all mutants were comparable to those of the WT. In the presence of ABA, *nua-3* was hypersensitive to ABA, while *snrk2.2 snrk2.3* and *abi5* mutants were insensitive to ABA as described previously. *nua-3 snrk2.2 snrk2.3* triple mutants and *nua-3 abi5* double mutants exhibited similar ABA insensitive phenotype as *snrk2.2 snrk2.3* and *abi5* mutants. These results indicate that NUA might act upstream of SnRK2.2/2.3 and AIB5 (Fig. 2).

### NUA physically interacts with SnRK2.2, SnRK2.3 and SnRK2.6

**Fig. 3:**
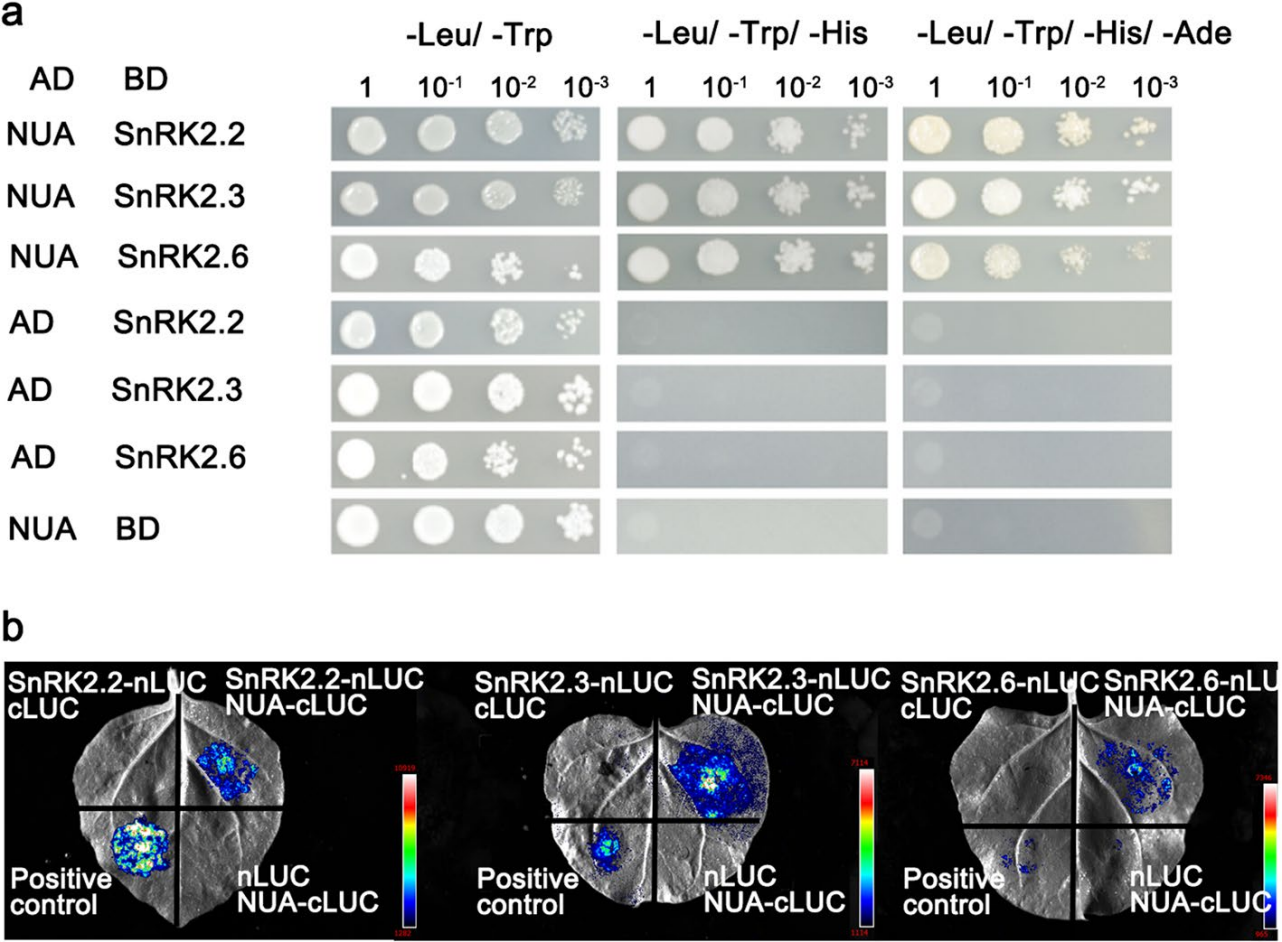
Assays for assessing NUA interactions with SnRK2.2, SnRK2.3 or SnRK2.6. **a** Yeast two-hybrid assay showing the interactions of NUA with SnRK2.2, SnRK2.3 or SnRK2.6. NUA was fused to the GAL4 AD. SnRK2.2, SnRK2.3 or SnRK2.6 was fused to the GAL4 BD. Serial 10-fold dilutions of yeast were spotted onto the plates. Left panels (-Leu/-Trp) show growth on plates selecting for the presence of the BD and AD plasmids. Middle panels (-Leu/-Trp/-His) and right panels (-Leu/-Trp/-His/-Ade) show growth on plates selecting for the presence of the BD and AD plasmids and the expression of reporter genes. The empty vectors were used in the negative controls. (BD: DNA binding domain; AD: activation domain). **b** LCI assay showing the interactions between NUA and SnRK2s in tobacco cells. The nLUC and cLUC derivative constructs were transformed into *A. tumefaciens* and then co-infiltrated into tobacco (*N. benthamiana*). The LUC signals were collected 48–72 h after infiltration. Empty cLUC and nLUC vectors were used as negative controls. The experiments were carried out with three independent biological repeats. (nLUC: N-terminal part of LUC; cLUC: C-terminal part of LUC)

SnRK2.2s are known to act upstream of ABI5 in the ABA signaling pathway (Lopez-Molina et al. 2001; Fujii et al. 2007), and since our previous genetic interaction data showed that NUA is epistatic to SnRK2s and ABI5 (Fig. 2), we speculated that NUA might physically interact with SnRK2.2, SnRK2.3 and/or SnRK2.6. To test this hypothesis, we carried out yeast two-hybrid (Y2H) assays. NUA fused with AD domain (NUA-AD) and SnRK2.2/2.3/2.6 fused with BD domain (SnRK2.2/2.3/2.6-BD) were co-transformed into AH109 yeast cells. Their physical interactions were then determined by examining cell growth on selective media. All colonies developed normally on the medium without tryptophan and leucine (–Trp/–Leu). On –Trp/–Leu/–His 3AT and –Trp/–Leu/–His/–Ade plates, only SnRK2s appeared to have interaction with NUA whereas the negative controls failed to survive (Fig. 3a). These results indicated that NUA can directly interact with SnRK2.2, SnRK2.3 and SnRK2.6 in yeast cells. Because there are ten members of the SnRK2 protein kinase family in Arabidopsis, we also tested the interactions between NUA and other SnRK2 kinases, and no interactions were observed among other SnRK2 kinases (Fig. S3).

To determine whether NUA interacts with SnRK2s *in planta*, we performed split-luciferase complementation assays using *Nicotiana benthamiana* leaves. The coding sequences of NUA and SnRK2.2/2.3/2.6 were cloned in-frame with those of nLUC and cLUC, respectively. The fusion plasmids were then transformed into *Agrobacterium*. LUC activity was then detected in the leaf area two days after transformation. Our results showed that NUA interacts with SnRK2.2/2.3/2.6 (Fig. 3b). Together, our Y2H and LCI assays indicated that NUA can physically interact with SnRK2.2/2.3/2.6 to regulate ABA signaling.

### ESD4 also interacts with SnRK2.2, SnRK2.3 and SnRK2.6 to negatively regulate ABA signaling

**Fig. 4:**
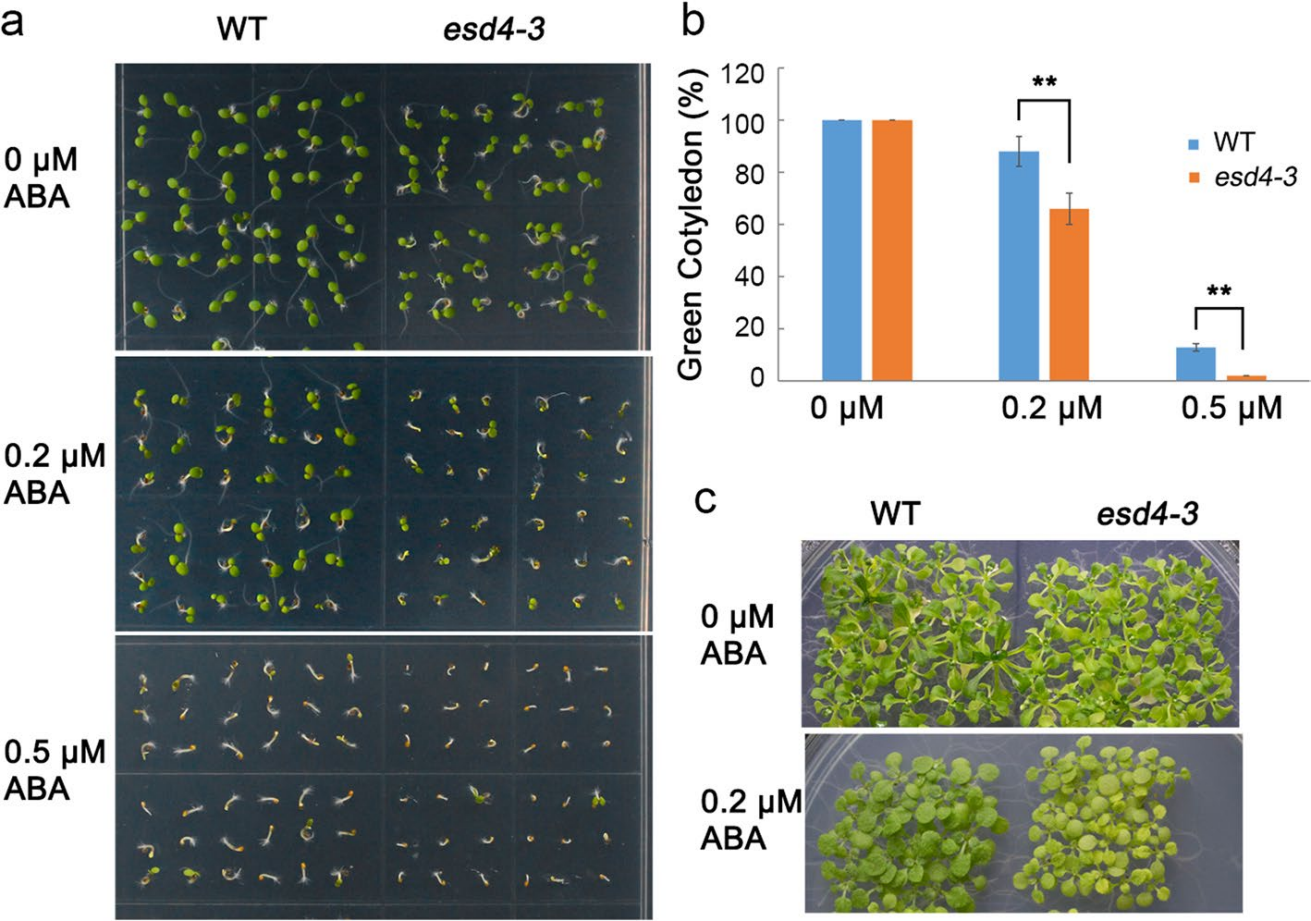
The *esd4-3* mutant is hypersensitive to ABA. **a** Phenotypic analyses of the wild type (WT) and *esd4-3* mutant treated by 0.2 or 0.5 μM ABA. The images were taken at Day 5 after the end of vernalization. **b** Seedling greening rates of *esd4-3* and WT plants. The ratios were counted at Day 5 after the end of vernalization. At least 30 seeds were counted in each replicate. Data are shown as means ± SD of three independent experiments. * *P* < 0.05 (Student's t-test). **c** The post-germination phenotype of *esd4-3* treated with ABA. The WT and *esd4-3* mutant seeds were germinated on MS medium or MS medium containing 0.2 μM ABA, and the pictures were taken 3 weeks after germination

It is known that NUA physically interacts with SUMO protease ESD4; and their loss-of-function mutants displayed similar phenotypes which include early flowering, stunted growth, mRNA accumulation in the nuclei, increased SUMO conjugates and decreased free SUMO (Xu et al. 2007). Based on these findings, we speculated that ESD4 may have similar roles in the ABA signaling as NUA. We thus obtained *esd4-3* mutant to test this hypothesis. We first counted the green cotyledon ratios at Day 5 after the end of vernalization. The cotyledon greening rates of *esd4-3* were similar with those of the WT under normal condition. However, when germinated on MS medium containing 0.2 μM or 0.5 μM ABA, *esd4-3* showed greater sensitivity to ABA (Fig. 4a, b). The growth of *esd4-3* seedling was also inhibited more by ABA compared to the WT (Fig. 4c). These data indicated that ESD4 can act as a negative regulator during seed germination and post-germination stages in response to ABA.

**Fig. 5:**
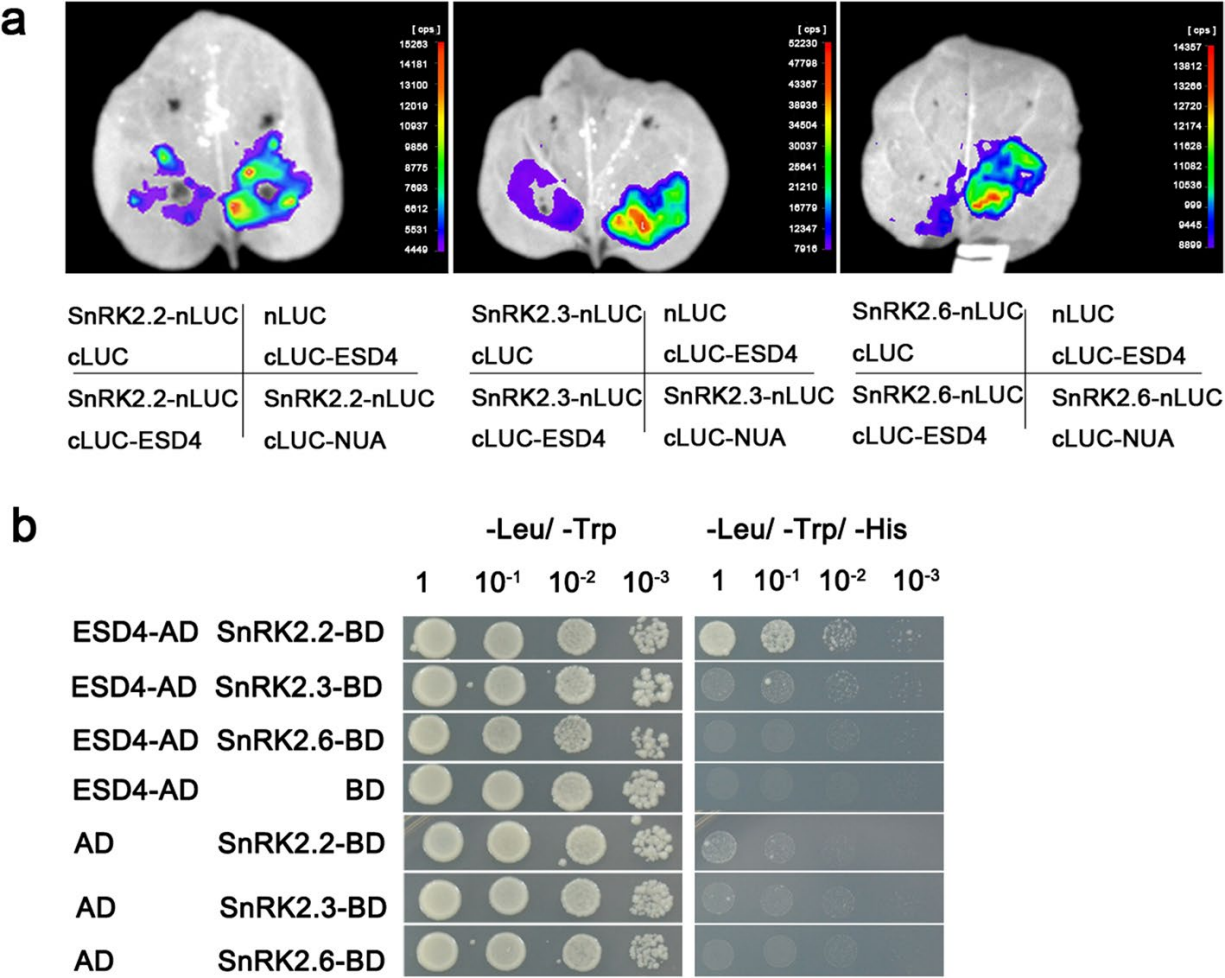
ESD4 protein physically interacts with SnRK2.2, SnRK2.3 and SnRK2.6. **a** ESD4 interacts with SnRK2s in a split firefly luciferase complementation assay. ESD4 fused to the C-terminus of LUC (cLUC-ESD4) was co-expressed with a SnRK2.2/2.3/2.6 fused N-terminus of LUC (nLUC- SnRK2.2/2.3/2.6) in *Nicotiana benthamiana* leaves. Images were collected 2 days after infiltration. Lower rows show the combinations of agrobacteria containing the indicated plasmids used to co-infiltrate into different leaf regions shown at upper rows. The combinations of cLUC-NUA and SnRK2s-nLUC were used as positive controls. **b** Yeast two-hybrid analysis of interactions between ESD4 and SnRK2s. ESD4 was fused to the GAL4 AD. Members of SnRK2 family were respectively fused to the GAL4 BD. Clones containing each combination of bait and prey vectors were cultured on nonselective media (-Trp/-Leu) and selective media (-Leu/-Trp/-His + 30 μM 3-AT). (BD: DNA binding domain; AD: activation domain)

Moreover, we investigated whether ESD4 can interact with SnRK2s by performing split-LUC complementation assays using cLUC-NUA and SnRK2s-nLUC as positive controls. Our results showed that ESD4 might have interaction with SnRK2s in planta as LUC signals were detected to be weaker than those in the NUA-SnRK2s (Fig. 5a). Furthermore, our Y2H results showed that only SnRK2.2 can directly interact with ESD4 (Fig. 5b). These results together suggested that NUA can act as an adaptor that links ESD4 protease with SnRK2s, then ESD4 and NUA form a large complex with SnRK2s.

### NUA and ESD4 affect the stability of SnRK2s

**Fig. 6:**
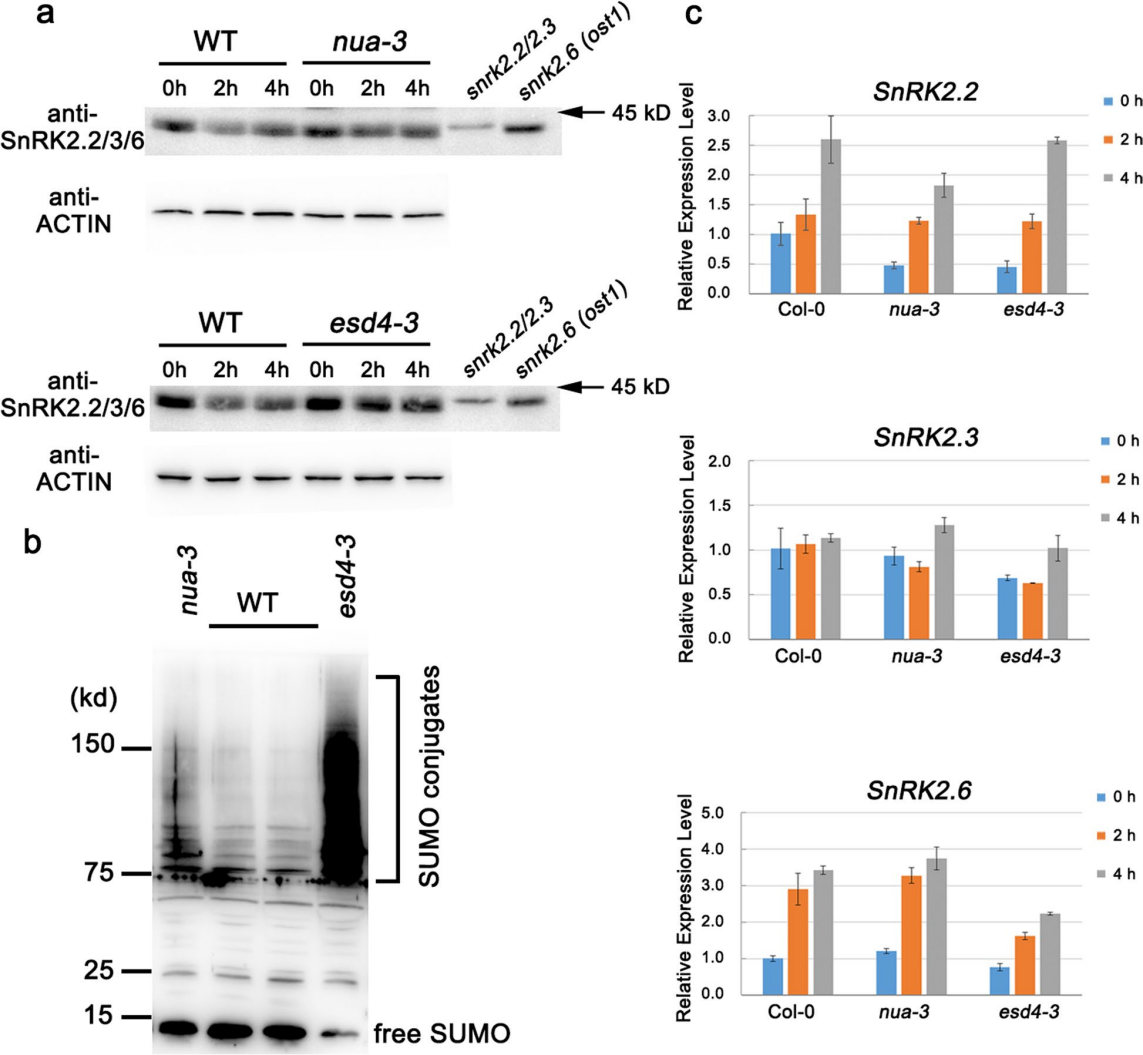
Protein abundance and transcription levels of SnRK2s in the *nua-3* and *esd4-3* mutants upon ABA treatment. **a** NUA and ESD4 positively regulate ABA-induced degradation of SnRK2s. Seeds were sown on 1/2 MS plates. 7-DAG seedlings were treated with 50 uM ABA for the indicated time. Total protein was extracted from the treated seedlings, and immunoblot assays were performed with anti-SnRK2.2/2.3/2.6 and ACTIN antibodies. The *snrk2.2/2.3* and *snrk2.6* (*ost1-3*) mutants were used as molecular weight references. **b** Accumulation of SUMO Conjugates in *nua-3* and *esd4-3*. Total protein was extracted from 14 d-old seedlings of the wild type, *nua-3* and *esd4-3*. 20 μg proteins were loaded onto an SDS-PAGE gel, and the immunoblot was probed with anti-SUMO1 antibody. **c** 7-DAG seedlings were treated by 50 μM exogenous ABA for 2 h and 4 h, and then the plants were collected for RNA extraction and Real-time quantitative PCR (RT-qPCR) analysis. The *ACTIN2* gene was used as an internal control. The expression levels of the indicated genes in WT were set to 1. Data are shown as means ± SD of three independent experiments

Previous research has reported that E3 ubiquitin ligases affect the stability of SnRK2s, while SUMOylation modification regulates protein stability (Miura et al. 2009; Zheng et al. 2012; Cheng et al. 2017). NUA has been reported to couple with ESD4 SUMO protease to participate SUMOylation (Xu et al. 2007). Since physical interactions were observed between NUA and SnRK2s in our study, we wanted to determine whether NUA and ESD4 can affect the stability of SnRK2s to mediate ABA signaling. We thus detected the protein levels of SnRK2s in 7-d-old *nua-3 and esd4-3* seedlings that have been treated with 50 μM ABA at indicated time points. Our western blotting results showed that SnRK2 proteins level decrease more gradually in the *nua-3* and *esd4-3* mutants relative to WT. The proteins level of *esd4-3* mutant treated with ABA was obviously higher than that of WT. In terms of contrast, the SnRK2s in *nua-3* were more stable than WT only after 2-h ABA treatment (Fig. 6a). To test if ESD4 is more important than NUA, we investigated the SUMO conjugation pattern in *nua-3* and *esd4-3*, compared with the wild type. The results showed that both *nua-3* and *esd4-3* mutants lead to an increase in SUMO conjugates and decrease in free SUMO. But *esd4-3* mutants accumulated more SUMO conjugates and had less free SUMO than *nua-3* mutant, indicating that ESD4 played a major role in deSUMOylation, thus it is reasonable that SnRK2s in *esd4-3* were more stable than *nua-3* (Fig. 6a, b). To exclusive the affects of transcription, we detected the mRNA level of SnRK2s in the mutants upon 50 μM ABA treatment. Real-time quantitative PCR (RT-qPCR) analysis revealed that the expressions of *SnRK2.2* and *SnRK2.6* were induced by ABA, while the expression level of *SnRK2.3* had no obvious changes. Moreover, there were no significant differences between WT and the mutants after ABA treatment (Fig. 6c). These results indicated that the changes of protein level (Fig. 6a) were not due to the mRNA level. Combined with the previous studies, we conclude that NUA and ESD4 can affect the stability of SnRK2s in the ABA signaling pathway in Arabidopsis.

## Discussion

NUA is a large protein located on the nucleoplasmic side of the NPCs in Arabidopsis. NUA physically interacts with ESD4 SUMO protease to regulate SUMO-homeostasis, mRNA export and flowering (Xu et al. 2007). In this study, we uncovered a novel function of NUA acting as a negative regulator in ABA signaling.

Although previous reports have shown that SUMOylation modification participates in ABA signaling (Lois et al. 2003; Miura et al. 2009; Zheng et al. 2012), no studies have yet investigated the functions of NUA and ESD4 in the ABA signaling pathway. Here, we revealed that both *nua* and *esd4* mutants were hypersensitive to ABA in the germination and post-germination stages (Figs. 1, 4). The genetic interaction studies showed that NUA can act upstream of SRNK2s in ABA signaling (Fig. 2). The Y2H and LCI assays confirmed the physical interactions of NUA and SnRK2.2/2.3/2.6 (Fig. 3). The interactions between ESD4 and SnRK2s were observed to be weaker (Fig. 5a), so NUA might serve as an adaptor that links ESD4 SUMO protease and SnRK2s to negatively regulate SnRK2s stability. Based on these results, we speculate that SnRK2 kinases might be substrates of the ESD4 SUMO protease. SnRK2s have SUMO-binding ΨKXE motif(s) using SUMOplot (http://www.abgent.com/sumoplot) analyses and are nuclear localized (Fujita et al. 2009), which are consistent with previous evidence of most SUMOylated proteins are nucleoproteins containing at least one ΨKXE sequence (Miller et al. 2010). Further experimental evidence is needed to confirm SnRK2s can be SUMOylated in Arabidopsis. It has been shown that the level of high molecular weight SUMO conjugates increased in both *nua* and *esd4* mutants (Fig. 6b) (Murtas et al. 2003; Xu et al. 2007). It is possible that the level of SUMO-SnRK2 conjugates might also increase in *nua* and *esd4* mutants.

It was previously reported that SUMOylation modulates protein stability. For example, SIZ1 (SAP and Miz) is a principal SUMO E3 ligase in Arabidopsis. SIZ1 negatively controls ABA signaling through the SUMOylation of ABI5 and MYB30 transcription factors. The conjugation of SUMO to ABI5 represses its activity but prevents ABI5 from undergoing degradation (Miura et al. 2009), while SUMOylation of MYB30 stabilizes MYB30 upon ABA treatment (Zheng et al. 2012). SIZ1 is also involved in aluminum (Al) resistance by SUMOylation of the zinc-finger transcription factor SENSITIVE TO PROTON RHIZOTOXICITY 1 (STOP1). The STOP1 protein levels were decreased in *siz1* mutant (Fang et al. 2021; Mercier et al. 2021; Roy and Sadanandom 2021; Xu et al. 2021). Interestingly, the SUMOylation level of STOP1 is also regulated by ESD4 (Q. Fang et al. 2020). Our findings of NUA and ESD4 interacting with SnRK2s (Figs. 3, 5), combining with the protein stability results (Fig. 6a), suggested that NUA and its partner ESD4 SUMO protease can modulate SnRK2s stability mostly via affecting their SUMOylation levels.

Furthermore, it was reported that SUMOylation affects protein stability by regulating ubiquitination-mediated protein degradation (Lois et al. 2003; Wilkinson and Henley 2010; Fang et al. 2020; Mercier et al. 2021). The SUMO substrates, such as ABI5, MYB30 and STOP1 proteins described above, are also regulated by the ubiquitin-26S proteasome pathway (Lopez-Molina et al. 2003; Zheng et al. 2018; Zhang et al. 2019). SUMOylation and ubiquitination might act antagonistically to regulate the stability of proteins so SUMOylation proteins are mostly stable because SUMO modification can prevent the ubiquitin proteasome-mediated degradation of proteins. ABA has been reported to promote the degradation of SnRK2.3 via ubiquitin proteasome-mediated pathway (Cheng et al. 2017). In our current work, we detected SnRK2 proteins were more stable in *nua-3* and *esd4-3* than in the WT (Fig. 6), suggesting that SUMO modification represses ubiquitination-mediated degradation of SnRK2 kinases. Future studies are needed to examine the ubiquitination level of SnRK2s in *nua* and *esd4* mutants.

A previous report has revealed that *Oryza sativa* OVERLY TOLERANT TO SALT1 (OsOTS1), a SUMO protease in rice, negatively regulated ABA signaling by reducing the stability of OsbZIP23 transcription factor (Srivastava et al. 2017). This finding coincides with our results of how NUA and ESD4 may function in regulating Arabidopsis’ ABA signaling. We want to make a note that in one report, Arabidopsis SUMO protease 1 (ASP1) was shown to positively regulate ABA signaling by promoting MYB30 stability and inhibiting the degradation of ABI5 (Wang et al. 2018a, b, c). This contradiction shows that the underlying regulatory mechanisms of SUMO protease are distinct. In our study, we found that *abi5* rescued the germination phenotype of *nua* mutant (Fig. 2) due to the epistatic effect of NUA to SnRK2s as well as SnRK2s acting upstream of ABI5. However, it is also possible that NUA can directly modulate ABI5. Moreover, MYB30 might also be a substrate of NUA and ESD4. Therefore, it would be interesting to investigate the interactions between NUA, ABI5, and MYB30 in the future.

In conclusion, we demonstrated that NUA and ESD4 can play critical roles in response to ABA by regulating the stability of SnRK2s and mediating seed germination and seedling growth in Arabidopsis. This work lays a foundation for a further dissection of the mechanism by which SUMO modification regulates ABA signaling.

## Materials and methods

### Plant materials and growth conditions

*Arabidopsis thaliana* Columbia-0 ecotype was used as the wild type. The *NUA* T-DNA insertion lines *nua-2* (SALK_069922) and *nua-3* (SAIL_505_H11) were obtained from NASC. The *snrk2.2 snrk2.3 *double mutant and *abi5-1* were previously described (Fujii et al. 2007). The *esd4-3* (Fang et al. 2020) mutant seed was obtained from AraShare (https://www.arashare.cn/index/). The genotyping primer sequences used are listed in Supplemental Table 1.

For the seed germination assay, seeds were surface-sterilized and then grown on 1/2 MS medium with or without ABA containing 1% (w/v) sucrose and 1% agar. The plates were stratified in darkness for 2 days at 4°C and then transferred to a light incubator at 22°C under a 16-h light /8-h dark photoperiod.

### RNA extraction and Quantitative RT-PCR

Total RNA was extracted from 7-day-old seedlings with TRIzol reagent ((Life, Invitrogen). The RNA was reverse transcribed to cDNA with MMLV reverse transcriptase (Yeasen). Quantitative RT-PCR was performed on an IQ5 Multicolor Real-Time PCR Detection system, using qPCR SYBR Green Master Mix (No Rox, Yeasen). *ACTIN2* was used as an internal control. For each genotype, three different samples were used for three replicates. The primers used for qRT-PCR analysis are listed in Supplemental Table 1.

### Yeast Two-Hybrid Assay

The *NUA* or *ESD4* coding region was cloned in-frame between the NdeI and BamHI sites of pGADT7. The full-length coding sequences of SnRK2s were cloned into pGBKT7 (Hou et al. 2016). Plasmid DNAs of bait and prey constructs were transformed into the *S. cerevisiae* strain *AH109*. The protein interactions were assayed on selection media plates (-Leu/-Trp/-His or -Leu/-Trp/-His/-Ade).

### Split-LUC Complementation Analysis

The split-LUC complementation analysis was performed as previously described (Zhou et al. 2018). The CDSs of *SnRK2.2*, *SnRK2.3* and *SnRK2.6* were cloned into pCAMBIA1300-nLUC. The CDSs of *NUA* and *ESD4* were cloned into pCAMBIA1300-cLUC. *A. tumefaciens* (strain GV3101) cells containing above plasmids were injected into *N. benthamiana* leaves. The empty cLUC and nLUC vectors were used as negative controls. After infiltration, plants were kept in the greenhouse for 40 to 48 hr. *N. benthamiana* leaves were incubated with 1 mM D-luciferin (Goldbio) in darkness for 3 min and then subjected for LUC signal detection by using Chemiluminescent Imaging System.

### Determination the protein levels of SnRK2s

The 7-d-old seedlings were treated with ABA at the indicated times. The seedlings were grounded into fine powder using liquid nitrogen. Proteins were extracted with extraction buffer [20 mM Tris-HCl (pH 7.4), 300 mM NaCl, 5 mM MgCl_2_ (pH 8.0), 5 mM DTT, 0.5% (v/v) Nonidet P-40, 50 μM MG132, 0.05% [w/v] SDS, and 1 × complete protease inhibitor mixture]. The protein extracts were spun for 30 min at 16,000 g at 4 °C. Protein concentration was measured using the Bio-Rad Protein Assay reagent (Bio-Rad, USA). The total proteins were separated by 10% (w/v) SDS-PAGE, and the SnRK2 proteins were analyzed by standard immunoblot using anti-SnRK2.2/2.3/2.6 (AS142783, Agrisera) and anti-ACTIN (343560, abmart) antibodies.

### Immunoprecipitation and Sumoylation Assays

The SUMOylation analysis was performed as previously described (Xu et al. 2007). Total protein was extracted from 14-d-old seedlings grown on 1/2 MS medium. Plant tissues (0.2 g) were extracted with extraction buffer. Protein concentration was measured using the Bio-Rad Protein Assay reagent (Bio-Rad, USA), and 20 μg protein was separated by SDS-PAGE, transferred to PVDF membrane, probed with anti-SUMO1 antibody (ab5316, Abcam).

### Chlorophyll measurement

Chlorophyll was extracted from leaves with 95% ethyl alcohol in water, and the absorption of the extracted solutions was measured at 663 and 645 nm using a microplate reader. The chlorophyll a concentration was calculated as (12.7×OD_663_-2.69×OD_645_)× dilution fold /fresh weight. The chlorophyll b concentration was calculated as (22.9× OD_645_-4.68 × OD_663_)×dilution fold /fresh weight.

## Supplementary Information

**Additional file 1: Fig. S1.** *nua-3* and *esd4-3* mutants were less sensitive to salt stress than WT. a Seeds were germinated on MS medium and grown for 3 d before being transferred to MS medium or MS medium containing 150 mM NaCl. The pictures were taken 5 days after transfer. The arrows indicate albino seedlings under salt stress. **b** Effect of salt stress on survival rate (seedlings with green cotyledons). The survival rate of WT was set to 100%. Data are shown as means ± SD of three independent experiments. ** *P* < 0.01 (Student's t-test). **c** Effect of salt stress on total chlorophyll content. The chlorophyll content of WT, *nua-3* or *esd4-3* mutant on MS medium was set to 100%. **P < 0.01 (Student's t-test).

**Additional file 2: Fig. S2.** Expression of ABA-responsive genes in *nua-3* mutant and wild type. 7-DAG seedlings were treated by 20 μM exogenous ABA for 2 h and 5 h, and then the plants were collected for RNA extraction and RT-qPCR analysis. The *ACTIN2* gene was used as an internal control. The expression levels of the indicated genes in WT were set to 1. Data are shown as means ± SD of three independent experiments.

**Additional file 3: Fig. S3.** Physical interactions between NUA and SnRK2.4, SnRK2.5, SnRK2.7, SnRK2.8, SnRK2.9, SnRK2.10 were not detected by yeast two-hybrid. NUA was fused to the GAL4 AD. Members of SnRK2 family were respectively fused to the GAL4 BD. Clones containing each combination of bait and prey vectors were cultured on nonselective media (-Trp/-Leu), selective media (-Leu/-Trp/-His, -Leu/-Trp/-His/-Ade or -Leu/-Trp/-His/-Ade + X-α-gal). Yeast transformed with the SnRK2.2 bait plasmid and the NUA prey plasmid were used as positive controls. (BD: DNA binding domain; AD: activation domain).

**Additional file 4: Supplemental Table 1**. Primers used in this article.

## Data Availability

Data are contained within the article or Supplementary Material.

## Abbreviations

ABA: Abscisic acid
ABI5: ABA insensitive 5
ARK: Abiotic stress-responsive Raf-like kinase
ASP1: Arabidopsis SUMO protease 1
AtPP2-B11: Arabidopsis phloem protein 2-B11
BIN2: Brassinosteroid insensitive 2
ESD4: Early short days 4
HOS15: High osmotic stress 15
NPC: Nuclear pore complex
NUA: Nuclear pore anchor
OST1: Open Stomata 1
OTS1: Overly tolerant to salt 1
PP2C: Protein phosphatases 2C
PYL: PYR1-like
PYR1: Pyrabactin resistance 1
RAFs: Raf-like kinases
RCAR: Regulatory components of ABA receptors
SAE: SUMO-activating enzyme
SCE1: SUMO E2 conjugating enzyme
SIZ1: SAP and Miz
SnRK2: SNF1-related protein kinase 2s
STOP1: Sensitive to proton hizotoxicity 1
Y2H: Yeast two-hybrid

## References

[CR1] Ali A, Kim JK, Jan M, Khan HA, Khan IU, Shen M, Park J, Lim CJ, Hussain S, Baek D, Wang K, Chung WS, Rubio V, Lee SY, Gong Z, Kim WY, Bressan RA, Pardo JM, Yun DJ (2019). Rheostatic control of ABA signaling through HOS15-mediated OST1 degradation. Mol Plant.

[CR2] Cai Z, Liu J, Wang H, Yang C, Chen Y, Li Y, Pan S, Dong R, Tang G, Barajas-Lopez Jde D, Fujii H, Wang X (2014). GSK3-like kinases positively modulate abscisic acid signaling through phosphorylating subgroup III SnRK2s in *Arabidopsis*. Proc Natl Acad Sci U S A.

[CR3] Cheng C, Wang Z, Ren Z, Zhi L, Yao B, Su C, Liu L, Li X (2017). SCFAtPP2-B11 modulates ABA signaling by facilitating SnRK2.3 degradation in *Arabidopsis thaliana*. PLoS Genet.

[CR4] Cutler SR, Rodriguez PL, Finkelstein RR, Abrams SR (2010). Abscisic acid: emergence of a core signaling network. Annu Rev Plant Biol.

[CR5] Fang Q, Zhang J, Yang DL, Huang CF (2021). The SUMO E3 ligase SIZ1 partially regulates STOP1 SUMOylation and stability in *Arabidopsis thaliana*. Plant Signal Behav.

[CR6] Fang Q, Zhang J, Zhang Y, Fan N, van den Burg HA, Huang CF (2020). Regulation of aluminum resistance in *Arabidopsis* involves the SUMOylation of the Zinc finger transcription factor STOP1. Plant Cell.

[CR7] Fujii H, Verslues PE, Zhu JK (2007). Identification of two protein kinases required for abscisic acid regulation of seed germination, root growth, and gene expression in *Arabidopsis*. Plant Cell.

[CR8] Fujii H, Zhu J-K (2009). *Arabidopsis* mutant deficient in 3 abscisic acid-activated protein kinases reveals critical roles in growth, reproduction, and stress. Proc Natl Acad Sci U S A.

[CR9] Fujita Y, Nakashima K, Yoshida T, Katagiri T, Kidokoro S, Kanamori N, Umezawa T, Fujita M, Maruyama K, Ishiyama K, Kobayashi M, Nakasone S, Yamada K, Ito T, Shinozaki K, Yamaguchi-Shinozaki K (2009). Three SnRK2 protein kinases are the main positive regulators of abscisic acid signaling in response to water stress in *Arabidopsis*. Plant Cell Physiol.

[CR10] Geiss-Friedlander R, Melchior F (2007). Concepts in sumoylation: a decade on. Nat Rev Mol Cell Biol.

[CR11] Hickey CM, Wilson NR, Hochstrasser M (2012). Function and regulation of SUMO proteases. Nat Rev Mol Cell Biol.

[CR12] Hou YJ, Zhu Y, Wang P, Zhao Y, Xie S, Batelli G, Wang B, Duan CG, Wang X, Xing L, Lei M, Yan J, Zhu X, Zhu JK (2016). Type one protein phosphatase 1 and its regulatory protein inhibitor 2 negatively regulate ABA signaling. PLoS Genet.

[CR13] Jacob Y, Mongkolsiriwatana C, Veley KM, Kim SY, Michaels SD (2007). The nuclear pore protein AtTPR is required for RNA homeostasis, flowering time, and auxin signaling. Plant Physiol.

[CR14] Kim DY, Scalf M, Smith LM, Vierstra RD (2013). Advanced proteomic analyses yield a deep catalog of ubiquitylation targets in *Arabidopsis*. Plant Cell.

[CR15] Lin Z, Li Y, Zhang Z, Liu X, Hsu CC, Du Y, Sang T, Zhu C, Wang Y, Satheesh V, Pratibha P, Zhao Y, Song CP, Tao WA, Zhu JK, Wang P (2020). A RAF-SnRK2 kinase cascade mediates early osmotic stress signaling in higher plants. Nat Commun.

[CR16] Lois LM, Lima CD, Chua N-H (2003). Small ubiquitin-like modifier modulates abscisic acid signaling in *Arabidopsis*. Plant Cell.

[CR17] Lopez-Molina L, Mongrand S, Chua NH (2001). A postgermination developmental arrest checkpoint is mediated by abscisic acid and requires the ABI5 transcription factor in *Arabidopsis*. Proc Natl Acad Sci U S A.

[CR18] Lopez-Molina L, Mongrand S, Kinoshita N, Chua NH (2003). AFP is a novel negative regulator of ABA signaling that promotes ABI5 protein degradation. Genes Dev.

[CR19] Ma Y, Szostkiewicz I, Korte A, Moes D, Yang Y, Christmann A, Grill E (2009). Regulators of PP2C phosphatase activity function as abscisic acid sensors. Science.

[CR20] Mercier C, Roux B, Have M, Le Poder L, Duong N, David P, Leonhardt N, Blanchard L, Naumann C, Abel S, Cuyas L, Pluchon S, Nussaume L, Desnos T (2021). Root responses to aluminium and iron stresses require the SIZ1 SUMO ligase to modulate the STOP1 transcription factor. Plant J Cell Mol Biol.

[CR21] Miller MJ, Barrett-Wilt GA, Hua Z, Vierstra RD (2010) Proteomic analyses identify a diverse array of nuclear processes affected by small ubiquitin-like modifier conjugation in *Arabidopsis*. PNAS. 10.1073/pnas.1004181107/-/DCSupplemental 10.1073/pnas.1004181107 PMC2944710 20813957

[CR22] Miura K, Lee J, Jin JB, Yoo CY, Miura T, Hasegawa PM (2009). Sumoylation of ABI5 by the *Arabidopsis* SUMO E3 ligase SIZ1 negatively regulates abscisic acid signaling. Proc Natl Acad Sci U S A.

[CR23] Murtas G, Reeves PH, Fu YF, Bancroft I, Dean C, Coupland G (2003). A nuclear protease required for flowering-time regulation in *Arabidopsis* reduces the abundance of SMALL UBIQUITIN-RELATED MODIFIER conjugates. Plant Cell.

[CR24] Mustilli AC, Merlot S, Vavasseur A, Fenzi F, Giraudat J (2002). Arabidopsis OST1 protein kinase mediates the regulation of stomatal aperture by abscisic acid and acts upstream of reactive oxygen species production. Plant Cell.

[CR25] Nakashima K, Fujita Y, Kanamori N, Katagiri T, Umezawa T, Kidokoro S, Maruyama K, Yoshida T, Ishiyama K, Kobayashi M, Shinozaki K, Yamaguchi-Shinozaki K (2009). Three Arabidopsis SnRK2 protein kinases, SRK2D/SnRK2.2, SRK2E/SnRK2.6/OST1 and SRK2I/SnRK2.3, involved in ABA signaling are essential for the control of seed development and dormancy. Plant Cell Physiol.

[CR26] Park HJ, Kim WY, Park HC, Lee SY, Bohnert HJ, Yun DJ (2011). SUMO and SUMOylation in plants. Mol Cells.

[CR27] Roy D, Sadanandom A (2021). SUMO mediated regulation of transcription factors as a mechanism for transducing environmental cues into cellular signaling in plants. Cellu Mol Life Sci.

[CR28] Saruhashi M, Kumar Ghosh T, Arai K, Ishizaki Y, Hagiwara K, Komatsu K, Shiwa Y, Izumikawa K, Yoshikawa H, Umezawa T, Sakata Y, Takezawa D (2015). Plant Raf-like kinase integrates abscisic acid and hyperosmotic stress signaling upstream of SNF1-related protein kinase2. Proc Natl Acad Sci U S A.

[CR29] Srivastava AK, Zhang C, Caine RS, Gray J, Sadanandom A (2017). Rice SUMO protease overly tolerant to salt 1 targets the transcription factor, OsbZIP23 to promote drought tolerance in rice. Plant J Cell Mol Biol.

[CR30] Takahashi Y, Zhang J, Hsu PK, Ceciliato PHO, Zhang L, Dubeaux G, Munemasa S, Ge C, Zhao Y, Hauser F, Schroeder JI (2020). MAP3Kinase-dependent SnRK2-kinase activation is required for abscisic acid signal transduction and rapid osmotic stress response. Nat Commun.

[CR31] Wang H, Tang J, Liu J, Hu J, Liu J, Chen Y, Cai Z, Wang X (2018). Abscisic acid signaling inhibits brassinosteroid signaling through dampening the dephosphorylation of BIN2 by ABI1 and ABI2. Mol Plant.

[CR32] Wang K, He J, Zhao Y, Wu T, Zhou X, Ding Y, Kong L, Wang X, Wang Y, Li J, Song CP, Wang BS, Yang S, Zhu JK, Gong Z (2018a) EAR1 negatively regulates ABA signaling by enhancing 2C protein phosphatase activity. Plant Cell. 10.1105/tpc.17.00875 10.1105/tpc.17.00875 PMC5969277 29618630

[CR33] Wang Q, Qu GP, Kong X, Yan Y, Li J, Jin JB (2018). *Arabidopsis* small ubiquitin-related modifier protease ASP1 positively regulates abscisic acid signaling during early seedling development. J Integr Plant Biol.

[CR34] Wilkinson KA, Henley JM (2010). Mechanisms, regulation and consequences of protein SUMOylation. Biochem J.

[CR35] Xu J, Zhu J, Liu J, Wang J, Ding Z, Tian H (2021). SIZ1 negatively regulates aluminum resistance by mediating the STOP1-ALMT1 pathway in *Arabidopsis*. J Integr Plant Biol.

[CR36] Xu XM, Rose A, Muthuswamy S, Jeong SY, Venkatakrishnan S, Zhao Q, Meier I (2007). NUCLEAR PORE ANCHOR, the *Arabidopsis* homolog of Tpr/Mlp1/Mlp2/megator, is involved in mRNA export and SUMO homeostasis and affects diverse aspects of plant development. Plant Cell.

[CR37] Yates G, Srivastava AK, Sadanandom A (2016). SUMO proteases: uncovering the roles of deSUMOylation in plants. J Exp Bot.

[CR38] Zhang Y, Zhang J, Guo J, Zhou F, Singh S, Xu X, Xie Q, Yang Z, Huang CF (2019). F-box protein RAE1 regulates the stability of the aluminum-resistance transcription factor STOP1 in *Arabidopsis*. Proc Natl Acad Sci U S A.

[CR39] Zheng Y, Chen Z, Ma L, Liao C (2018). The ubiquitin E3 ligase RHA2b promotes degradation of MYB30 in abscisic acid signaling. Plant Physiol.

[CR40] Zheng Y, Schumaker KS, Guo Y (2012). Sumoylation of transcription factor MYB30 by the small ubiquitin-like modifier E3 ligase SIZ1 mediates abscisic acid response in *Arabidopsis thaliana*. Proc Natl Acad Sci U S A.

[CR41] Zhou Z, Bi G, Zhou JM (2018). Luciferase complementation assay for protein-protein interactions in plants. Curr Protoc Plant Biol.

