# Supplementary figures and images for: NUA and ESD4 negatively regulate ABA signaling during seed germination
Source: Stress Biol. 2022 Sep 13;2(1):38. doi: 10.1007/s44154-022-00062-1 (PMC10442006; doi:10.1007/s44154-022-00062-1)

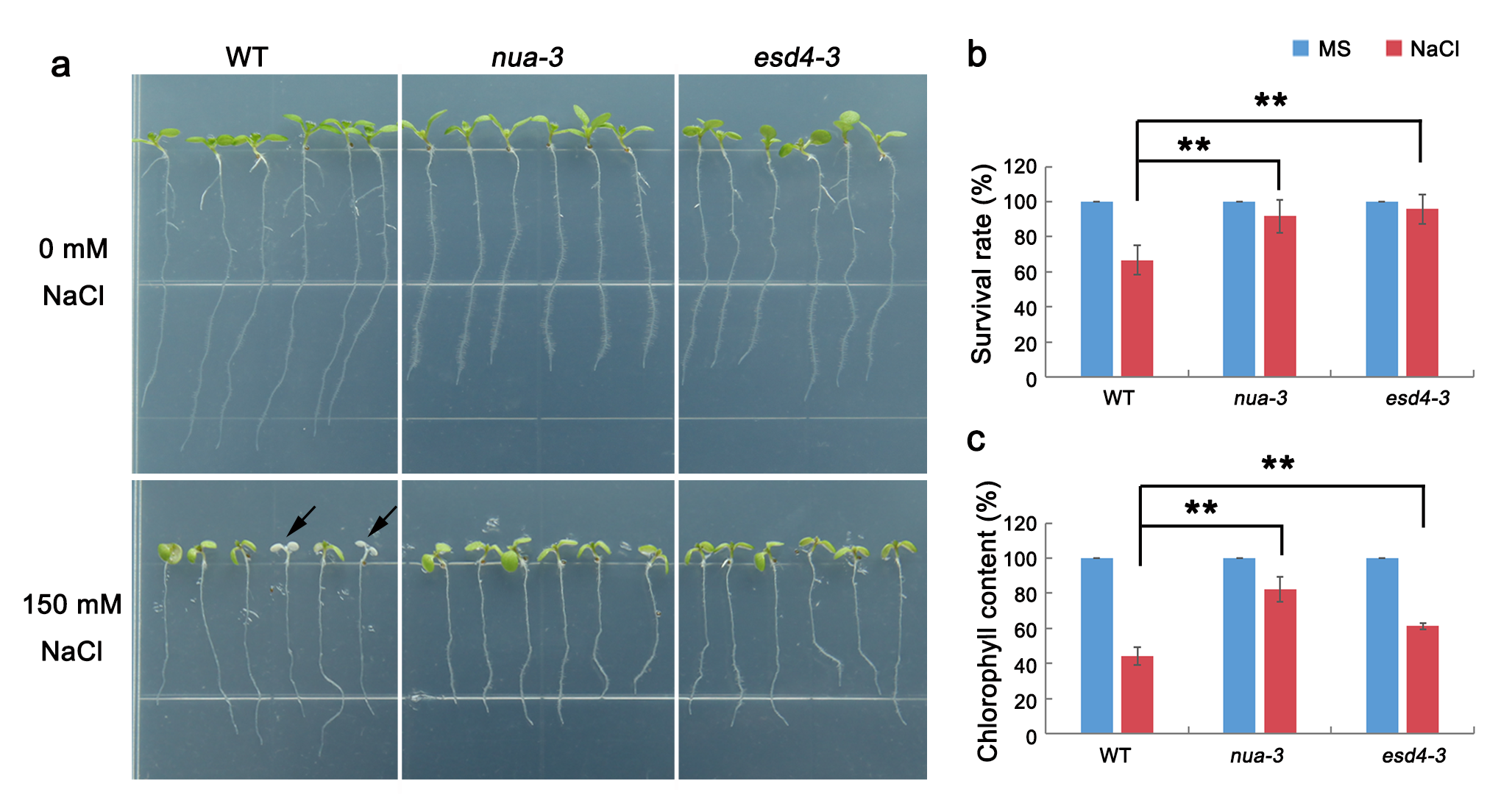

Supplement: Supplementary file 1 — Additional file 1: Fig. S1. nua-3 and esd4-3 mutants were less sensitive to salt stress than WT. a Seeds were germinated on MS medium and grown for 3 d before being transferred to MS medium or MS medium containing 150 mM NaCl. The pictures were taken 5 days after transfer. The arrows indicate albino seedlings under salt stress. b Effect of salt stress on survival rate (seedlings with green cotyledons). The survival rate of WT was set to 100%. Data are shown as means ± SD of three independent experiments. ** P < 0.01 (Student's t-test). c Effect of salt stress on total chlorophyll content. The chlorophyll content of WT, nua-3 or esd4-3 mutant on MS medium was set to 100%. **P < 0.01 (Student's t-test). [file 44154_2022_62_MOESM1_ESM.tif]

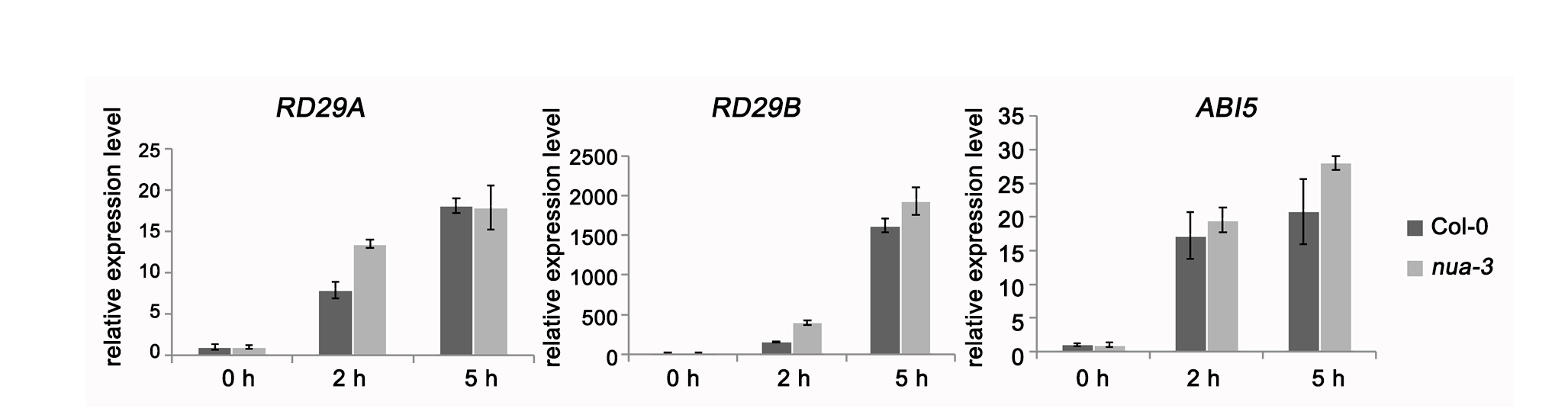

Supplement: Supplementary file 2 — Additional file 2: Fig. S2. Expression of ABA-responsive genes in nua-3 mutant and wild type. 7-DAG seedlings were treated by 20 μM exogenous ABA for 2 h and 5 h, and then the plants were collected for RNA extraction and RT-qPCR analysis. The ACTIN2 gene was used as an internal control. The expression levels of the indicated genes in WT were set to 1. Data are shown as means ± SD of three independent experiments. [file 44154_2022_62_MOESM2_ESM.tif]

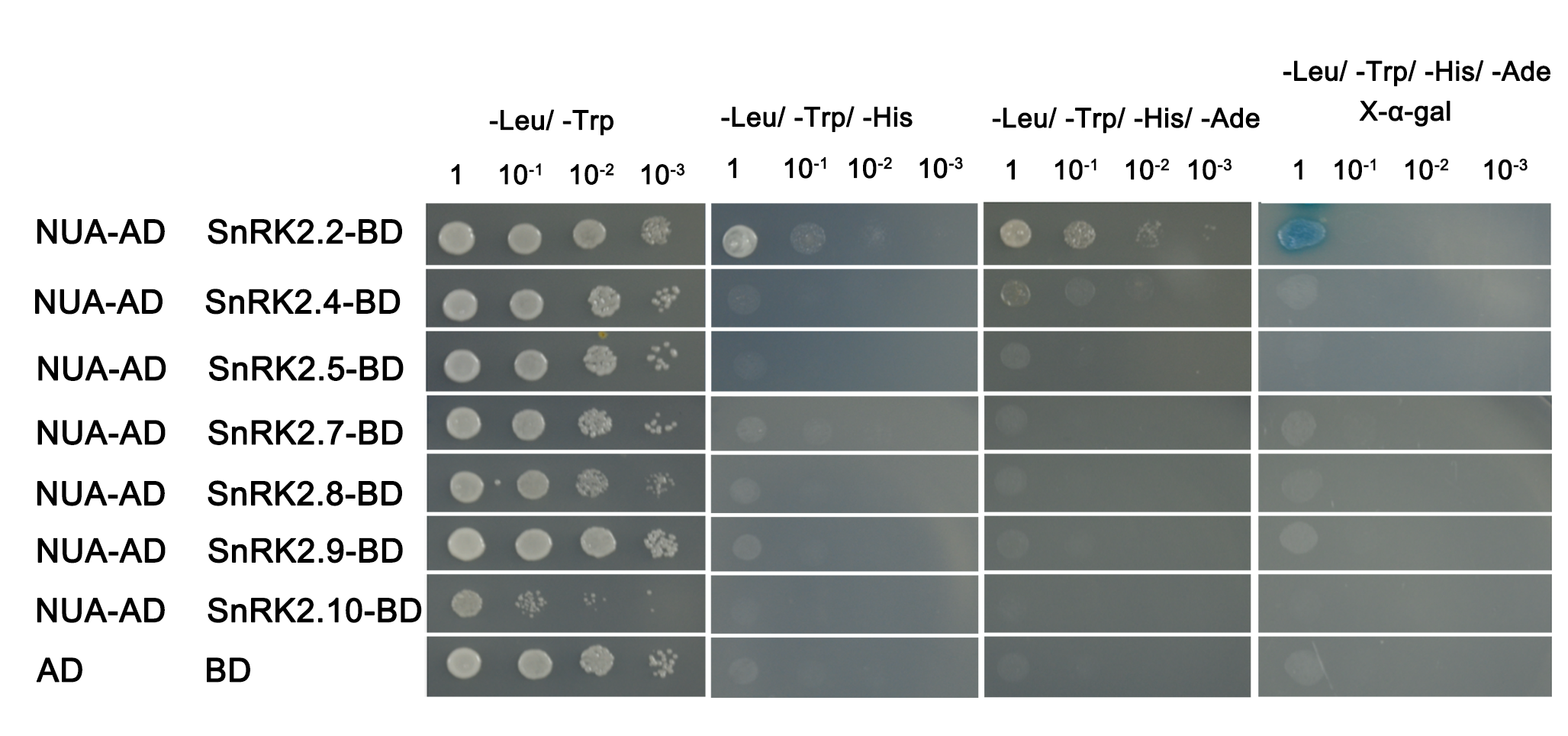

Supplement: Supplementary file 3 — Additional file 3: Fig. S3. Physical interactions between NUA and SnRK2.4, SnRK2.5, SnRK2.7, SnRK2.8, SnRK2.9, SnRK2.10 were not detected by yeast two-hybrid. NUA was fused to the GAL4 AD. Members of SnRK2 family were respectively fused to the GAL4 BD. Clones containing each combination of bait and prey vectors were cultured on nonselective media (-Trp/-Leu), selective media (-Leu/-Trp/-His, -Leu/-Trp/-His/-Ade or -Leu/-Trp/-His/-Ade + X-α-gal). Yeast transformed with the SnRK2.2 bait plasmid and the NUA prey plasmid were used as positive controls. (BD: DNA binding domain; AD: activation domain). [file 44154_2022_62_MOESM3_ESM.tif]
