# Supplementary material for: NUA and ESD4 negatively regulate ABA signaling during seed germination
Source: Stress Biol. 2022 Sep 13;2(1):38. doi: 10.1007/s44154-022-00062-1 (PMC10442006; doi:10.1007/s44154-022-00062-1)
Supplement: Supplementary file 4 — Additional file 4: Supplemental Table 1. Primers used in this article. [file 44154_2022_62_MOESM4_ESM.docx]

| **Primers for genotyping** |
| --- |
| SNRK2.2-F: CAAGACCATACATCTGCAAGCTGG  SNRK2.2-R: ACACCTTGATGTTTCTTCTGTGTG  GABI-TDNA-LB: TTGACCATCATACTCATTGCTGATCC  SNRK2.3-F: TTGGTTTTGAGTGTTCTGCTTTTG  SNRK2.3-R: ACCACATGACCATACATCTGCAA  JMLB1: GGCAATCAGCTGTTGCCCGTCTCACTGGTG  SnRK2.6-F: CATATCTTTAGACGAGGGGCC  SnRK2.6-R: GTGAGTGGTCCAATGGATTTG  JMLB1: GGCAATCAGCTGTTGCCCGTCTCACTGGTG  nua-2 F: GGCAATCAGCTGTTGCCCGTCTCACTGGTG  nua-2 R: TGAACGGGATAAGTTGGCGATGGA  LBa1: TGGTTCACGTAGTGGGCCATCG  nua-3 F: TGAACCAATTCCATACTGCATC  nua-3 R: ATTTCTCATGTTCAGTCTCCAG  SAIL_LB1: GCCTTTTCAGAAATGGATAAATAGCCTTGCTTCC  AS037-F: GCAAAATACATGGGTGATGAAG  AS037-R: ATCAGCTCGTAGCCTCAGAATC |
| **Primers for construction** |
| ESD4-cLUC-F: tacgcgtcccggggcggtaccATGGGTGCCGTAGCGATCAA  ESD4-cLUC-R: ACGAAAGCTCTGCAGGTCGACTCAATCAGCTCGTAGCCTCAGAA  ESD4-AD-F: GTACCAGATTACGCTCATATGATGGGTGCCGTAGCGATCAA  ESD4-AD-R: CAGCTCGAGCTCGATGGATCCATCAGCTCGTAGCCTCAGAA  NdeI-NUACDS-F: ACTCATATGCCCTTGTTTATGCCTGA  BamHI-NUACDSwsc-R: CGCGGATCC TCATGGTGGGCTCGGGGATTG  NUA-cLUC-F1: tacgcgtcccggggcggtaccATGCCCTTGTTTATGCCTGA  NUA-cLUC-R1: ACGAAAGCTCTGCAGGTCGACTCATGGTGGGCTCGGGGATTG  SNRK2.2-nLUC-F: cgagctcggtacccgggatccATGGATCCGGCGACTAATTCACC  SNRK2.2-nLUC-R: CGCGTACGAGATCTGGTCGACGAGAGCATAAACTATCTCTCCACTAC  SNRK2.3-nLUC-F: cgagctcggtacccgggatccATGGATCGAGCTCCGGTGACC  SNRK2.3-nLUC-R: CGCGTACGAGATCTGGTCGACGAGAGCGTAAACTATCTCTCCGCTAC  SNRK2.6-nLUC-F: cgagctcggtacccgggatccATGGATCGACCAGCAGTGAGTG  SNRK2.6-nLUC-R: CGCGTACGAGATCTGGTCGACCATTGCGTACACAATCTCTCCGCTAC |
| **Primers for qRT-PCR** |
| SnRK2.2 qPCR LP: AGGCAACGATTACGAACAGTCGG  SnRK2.2 qPCR RP: AGCCAATGCAGAGCCTTGACAC  SnRK2.3 qPCR LP: CACAGGACCGTTGGATATGCCG  SnRK2.3 qPCR RP: ACGAGCAACACCGAAATTACCAG  SnRK2.6 qPCR LP: ACCAGCAGTGAGTGGTCCAATG  SnRK2.6 qPCR RP: ACCGGAGCCAATATCCTTGACG  Actin_Q_Fw: ATGGAAGCTGCTGGAATCCAC  Actin_Q_Rv: TTGCTCATACGGTCAGCGATG  ABI5-Q-F: GAGAATGCGCAGCTAAAACA  ABI5-Q-R: GTGGACAACTCGGGTTCCTC  RD29A-F: TCCAGCATCGGAGGAAATTATTCCACCA  RD29A-R: TCCAGAAAGCAGAGAGACCGGAGT  RD29B-F: TGTCTTCTGACCACACCAAACCCATTG  RD29B-R: CCACCAGGAGCAAACGTCCTAGTCA |
